# A Case of Multiple Calcific Embolic Strokes in a Patient With a Porcelain Left Atrium

**DOI:** 10.7759/cureus.18585

**Published:** 2021-10-07

**Authors:** Abhinav Karan, Julien Feghaly, Hui Jun Guo, Temitope O Akinjogbin, Srinivasan Sattiraju

**Affiliations:** 1 Internal Medicine, University of Florida College of Medicine, Jacksonville, USA; 2 Cardiology, University of Florida College of Medicine, Jacksonville, USA

**Keywords:** 3d echo, cardiothoracic imaging, cardiothoracic, coronary artery angiography, clinical cardiology, embolic cva, mitral annular calcification, mitral valve annulus

## Abstract

Mitral annular calcification (MAC) commonly manifests as an incidental, asymptomatic finding that is associated with several cardiovascular risk factors, atherosclerosis, cardiovascular death, and all-cause mortality. Very rarely, patients with severe MAC can have extensive dystrophic calcification extending into the left atrial wall, termed porcelain left atrium. In this case report, we describe a patient who experienced multiple calcific acute embolic strokes in the setting of severe mitral annular calcification and porcelain left atrium. Our patient presented with multiple, small bilateral acute infarcts scattered throughout the cerebrum and cerebellum confirmed on magnetic resonance imaging (MRI). He was placed on continuous telemetry and underwent multimodal imaging with transthoracic and transesophageal echocardiography, carotid neck ultrasound (US), head and neck computed tomography angiogram (CTA), and cardiac MRI. There were no arrhythmic events detected on telemetry, and all imaging excluded left ventricular thrombi, aortic atheroma, carotid artery stenosis, intracardiac shunting, or large vessel stenosis. Noted on imaging, however, was severe mitral annular calcification with numerous, highly mobile calcific extensions and densely calcified plaque along the posterior left atrial wall, presumed to be the source of this patient's embolic stroke. Cardiac catheterization was significant for severe three-vessel disease requiring coronary artery bypass grafting, and our patient was subsequently discharged to outpatient follow-up on event monitoring and aspirin monotherapy. This case serves to highlight a previously unreported complication of calcific embolic stroke in severe MAC and porcelain left atrium, and highlight the need for further randomized controlled trials to determine the optimum management of these cases.

## Introduction

Mitral annular calcification (MAC) most commonly manifests as an incidental, asymptomatic finding among patients undergoing cardiac imaging and is rarely associated with calcification of the left atrium. Once present, these calcifications are significantly associated with several cardiovascular risk factors, atherosclerosis, cardiovascular death, and all-cause mortality [1]. In particularly severe cases, extensive dystrophic calcification can also involve the left atrial wall, leading to the seldom reported complication of porcelain left atrium [1]. The association between MAC and acute ischemic stroke has been well documented; however, its etiology is most commonly ascribed as a consequence of atherosclerotic disease, or valvular dysfunction and atrial fibrillation [2]. A case of calcific embolization has not yet been documented in the literature, and we aim to describe a case of multiple calcific embolic strokes in the setting of a porcelain left atrium.

## Case presentation

A 78-year-old Caucasian male with a past medical history of hyperlipidemia and gout presented with a one-day history of left-sided weakness and an altered mental status. Initial laboratory studies revealed elevated high-sensitivity troponin T values (0 hour: 131 ng/L; 3 hour: 127 ng/L; reference range: <22 ng/L) with a normal delta troponin value. Electrocardiogram was remarkable for left bundle branch block with left axis deviation, with no prior for comparison. Subsequent cerebral imaging with computed tomography (CT) scan of the brain and CT angiogram (CTA) of the head and neck was unremarkable; however, a CT perfusion study showed a possible perfusion abnormality in the cerebellum bilaterally. Telemetry revealed no underlying evidence of atrial fibrillation or other arrhythmias.

**Figure 1:**
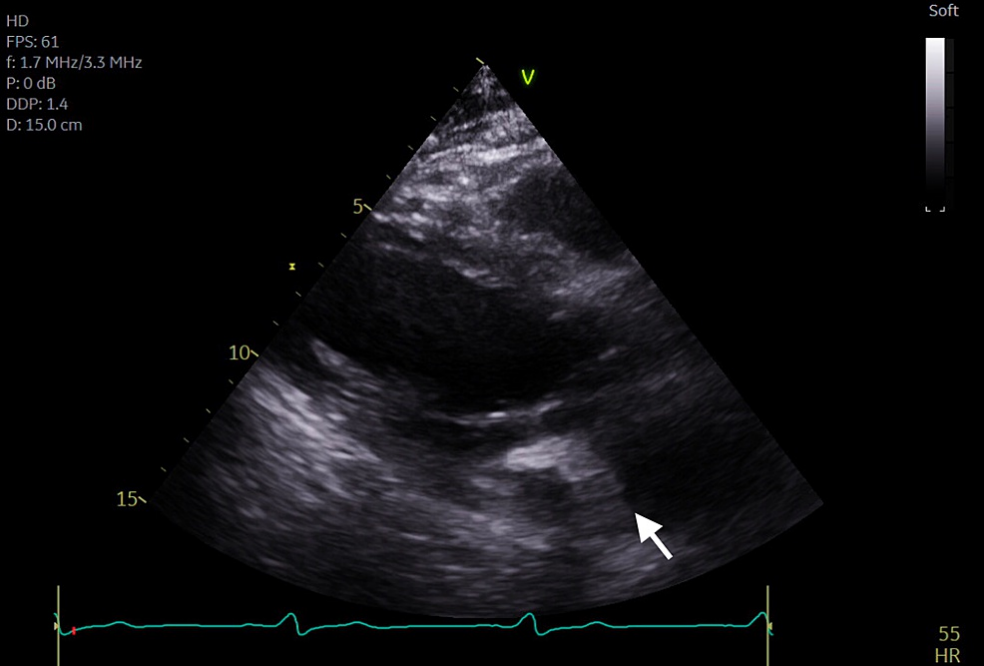
Transthoracic echocardiogram parasternal long axis view demonstrating severe mitral annular calcification with several mobile calcific extensions at lateral commissure imaged in both left atrium and left ventricle.

**Figure 2:**
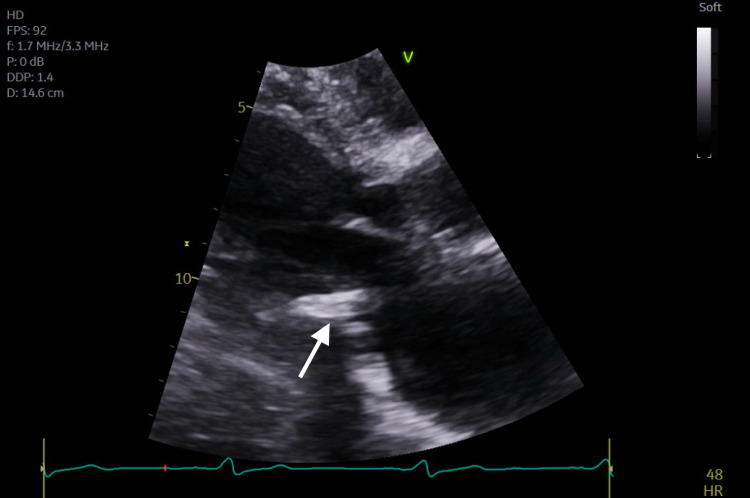
Transthoracic echocardiogram parasternal long axis view demonstrating severe mitral annular calcification with several mobile calcific extensions at lateral commissure imaged in both left atrium and left ventricle.

**Figure 3:**
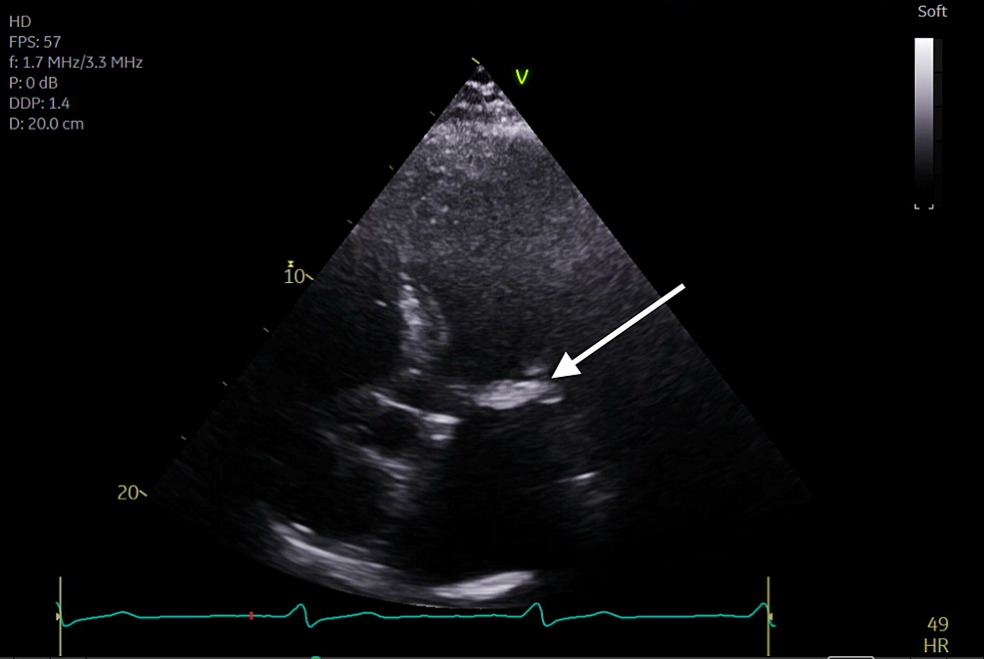
Transthoracic echocardiogram apical four-chamber view demonstrating severe mitral annular calcification.

**Figure 4:**
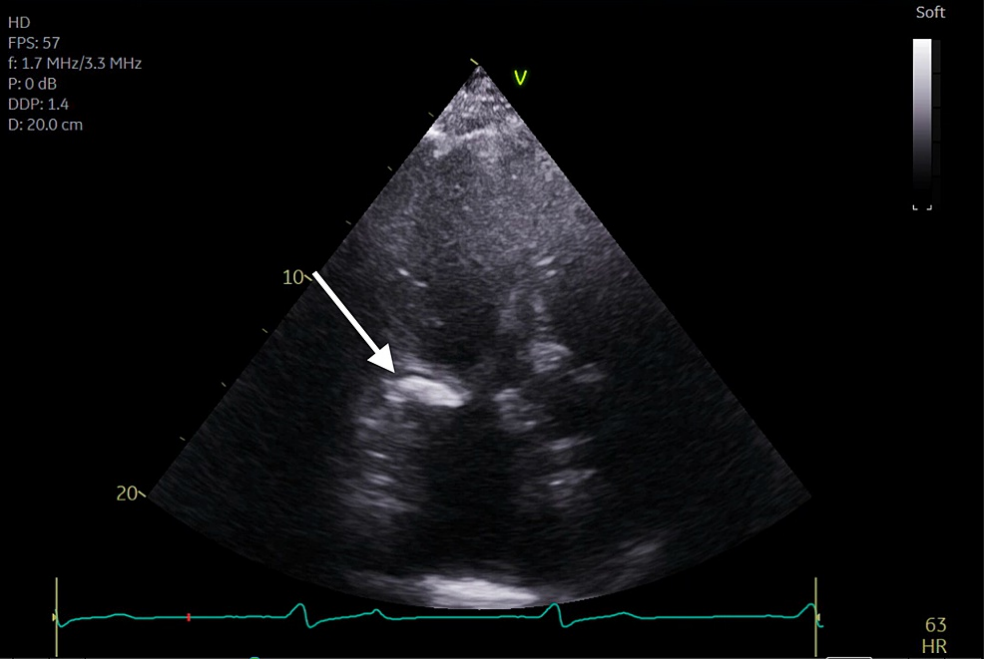
Transthoracic echocardiogram apical two-chamber view demonstrating severe mitral annular calcification.

**Figure 5:**
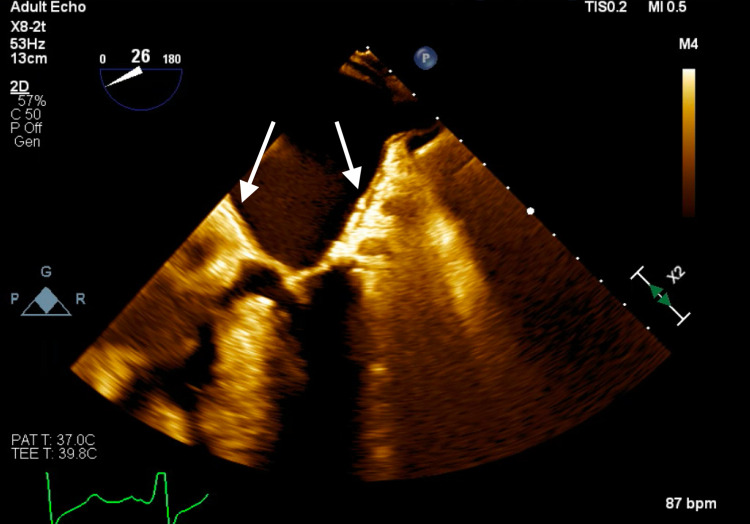
Transesophageal echocardiogram four-chamber view showing layering of highly echogenic material along the posterior left atrial wall.

**Figure 6:**
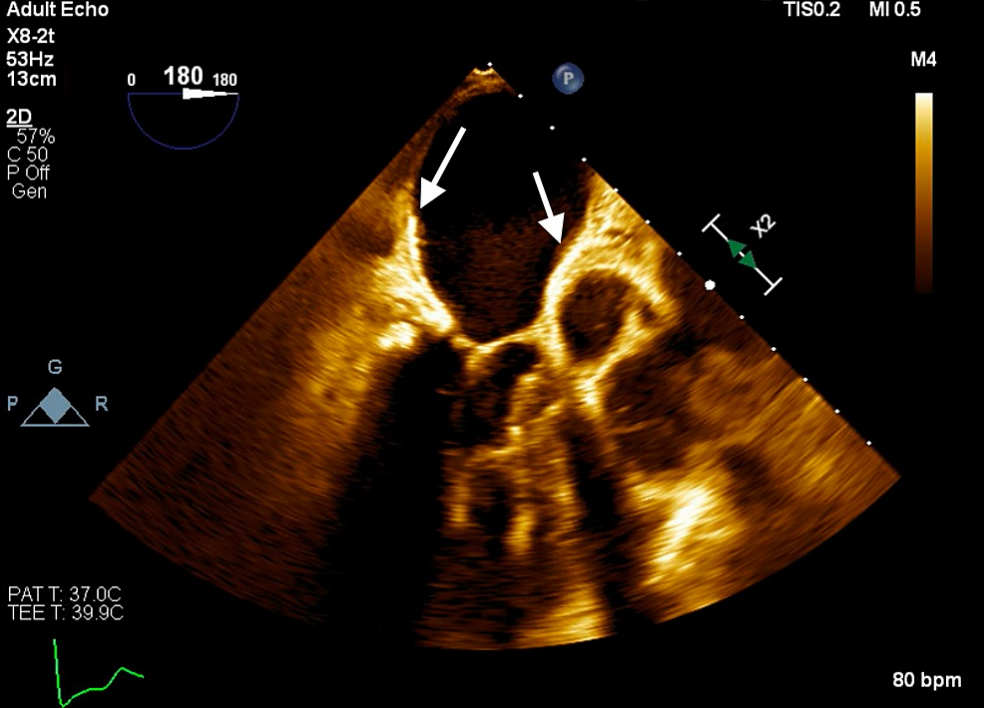
Transesophageal echocardiogram long axis view showing layering of highly echogenic material along the posterior left atrial wall.

**Figure 7:**
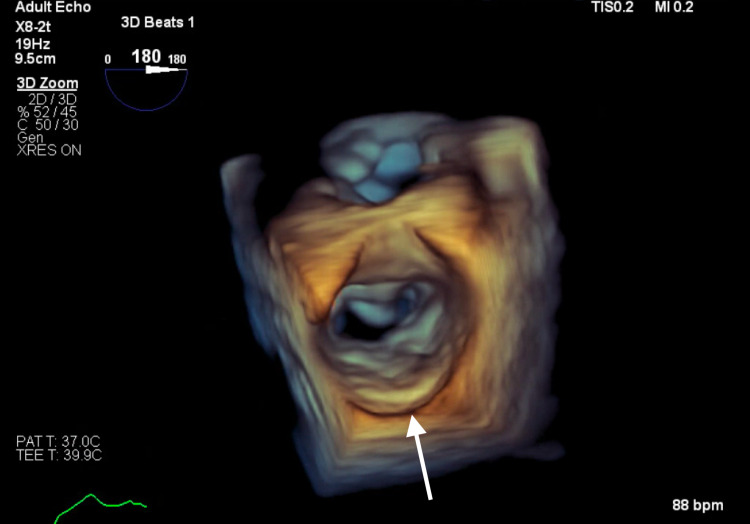
Transesophageal echocardiogram 3D surgeon view showing calcification of the left atrial posterior wall.

**Figure 8:**
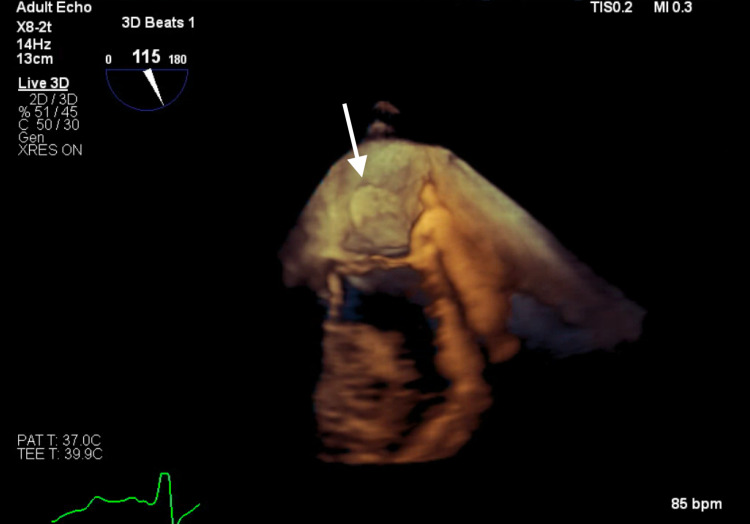
Transesophageal echocardiogram 3D volume data set cropped over the left atrium to reveal the extent of the left atrial wall calcification.

Subsequent magnetic resonance imaging (MRI) of the brain showed multiple, small bilateral acute ischemic infarcts scattered throughout the cerebrum, subcortical region, and periventricular regions, alongside a left ischemic cerebellar stroke. An embolic source was suspected, and a transthoracic echocardiogram (TTE) was ordered, which showed severe mitral annular calcification with several mobile, protruding calcific extensions at lateral commissure imaged in both left atrium and left ventricle, as can be seen in Figures 1-4 and Video 1. The patient subsequently underwent a transesophageal echocardiogram (TEE), which revealed no intracardiac shunt, cardiac thrombus, or aortic atheroma but better characterized the patient’s MAC, demonstrating highly calcified plaque along the posterior left atrial wall, as demonstrated in Figures 5-8 and Videos 2 and 3. A cardiac MRI scan further confirmed the calcific elements on the mitral valve annulus with involvement of the left atrium.

**Video 1:** Severe mitral annular calcification seen on parasternal long axis view of transthoracic echocardiogram, with several mobile protruding calcific extensions at lateral commissures in both left atrium and left ventricle.

**Video 2:** Transesophageal echocardiogram four-chamber view with Doppler demonstrating severe mitral annular calcification.

**Video 3:** Transesophageal echocardiogram 3D surgeon view showing extensive calcification of the left posterior atrial wall.

Subsequent carotid neck ultrasound (US) also ruled out carotid artery stenosis, and large vessel stenosis was excluded previously on the patient's head and neck CTA. Cardiac catheterization revealed severe multivessel coronary artery disease involving the distal left main, mid-left anterior descending, and distal right coronary artery, with significant calcification noted in the vasculature. The patient was then referred for cardiothoracic surgical evaluation for coronary artery bypass grafting. He was started on aspirin for possible cardioembolic stroke and discharged to outpatient follow-up with event monitoring, which thus far has not revealed any arrhythmias. 

## Discussion

While MAC is often an incidental finding discovered on cardiac imaging, found in as much as 42% of all patients receiving cardiac imaging for another indication, it carries essential prognostic significance for patients [1]. Calcifications of the mitral annulus often occur in the posterior aspect of the leaflet. However, multiple studies have shown that calcifications can extend beyond the leaflets and into other structures of the heart [1]. The Framingham Heart Study showed that MAC was associated with a 60% and 30% higher adjusted risk of cardiovascular and all-cause death, respectively [2]. Similarly, the Jackson Cohort of the Atherosclerotic Risk in Communities (ARIC) study showed a 132% increased adjusted risk of coronary artery disease [3], while the Cardiovascular Health Study (CHS) showed an 80% and a 30% higher adjusted risk for cardiovascular and all-cause death, respectively [4]. The prognostic importance of MAC therefore cannot be underestimated. One study estimated that with each 1-mm increase in MAC, there is an incident increase in the risk of cardiovascular disease, cardiovascular death, and all-cause mortality by approximately 10% [2]. There is an increased risk of acute ischemic strokes in patients with MAC with a relative adjusted risk of 2.1 and a continuous increased risk of acute ischemic strokes by 1.24 for each 1-mm increase in the thickness of calcification [4].

There exist several hypotheses on this association. MAC may relate to the progression of valvular dysfunction, which can lead to atrial fibrillation and subsequent stroke. Besides, it is also possible that the association between MAC, atrial fibrillation, and stroke is driven by changes in left atrial morphology and other mechanics that have yet to be elucidated [5]. Apart from the risk of atrial fibrillation, MAC's relationship to atherosclerosis is likely a major factor [4]. Among patients evaluated by TEE as part of the cerebral ischemia workup, MAC showed an association with both complex aortic atheroma, a high-risk emboligenic substrate [6], and ipsilateral large vessel stenosis, which can both represent underlying etiologies of stroke in MAC. In our patient, however, comprehensive evaluation with CT angiogram, TEE, TTE, and carotid neck US revealed none of these findings. Furthermore, our patient has demonstrated no arrhythmic events on outpatient event monitoring.

Our patient experienced acute multifocal strokes characteristic of an embolic etiology in the setting of severe MAC and severe left atrial calcification. We postulate, therefore, that the etiology of our patient’s presentation was embolization of calcific elements from mobile MAC and left atrial calcification. This case is unique, and there remains significant controversy and a lack of consensus on the management of MAC in such patient populations.

Current management strategies for MAC revolve around cardiovascular risk factor modification due to its significant association with atherosclerosis and cardiovascular disease. Despite this, there currently exist no trials on the impact of medical therapy on the progression of MAC. Drawing comparison, in patients with calcific aortic valve sclerosis, the Simvastatin and Ezetimibe in Aortic Stenosis (SEAS) trial showed that the addition of statin therapy had no benefit in mitigating the rate of progression of calcification on the aortic valve [7]. The management of MAC for modification of stroke risk has yet to be thoroughly explored. Currently, the only guideline for this is from the 2012 American College of Chest Physicians, who recommend the initiation of antithrombotic therapy with aspirin in patients with MAC and any incident of systemic embolism, transient ischemic attack, or stroke [8]. The management of MAC to decrease stroke risk should be an individualized treatment for each patient with the risk of increased cardiovascular disease being an important component.

## Conclusions

Mitral annular calcification represents a critical marker of underlying cardiovascular risk. Some patients may develop severe complications such as porcelain left atrium, which can lead to complications such as embolic calcific strokes as in our case. There currently exist no management guidelines on this unique patient population, and a highly individualized approach is required with a focus on randomized controlled trials to determine the standard of care. 
